# The complete chloroplast genome sequence of *Adenophora kayasanensis* Kitam. (Campanulaceae), an endemic to Korea

**DOI:** 10.1080/23802359.2021.2002212

**Published:** 2021-11-23

**Authors:** Kyung-Ah Kim, Yoo-Jung Park, Kyeong-Sik Cheon

**Affiliations:** a Environmental Research Institute, Kangwon National University, Chuncheon, South Korea; b Department of Biological Science, Sangji University, Wonju, South Korea

**Keywords:** Campanulaceae, *Adenophora*, Chloroplast genome, phylogeny

## Abstract

*Adenophora kayasanensis* Kitam., belonging to the family Campanulaceae, is an important species because it is used as a type of herbal medicine and is endemic to Korea. Here, we report the complete chloroplast genome sequence of *A. kayasanensis* as determine by means of Illumina high-throughput sequencing. The complete cp genome was 169,433 bp in length, containing a large single copy (LSC) of 123,110 bp and a small single copy (SSC) of 8619 bp, which were separated by a pair of 29,085 bp inverted repeats (IRs). A total of 112 unique genes were annotated, consisting of 78 protein-coding genes, 30 *tRNA* genes, and four *rRNA* genes. The overall GC content is 37.7%. A maximum-likelihood (ML) tree based on 76 protein-coding genes indicated that *A. kayasanensis* is closely related to *Adenophora racemosa*. This newly sequenced chloroplast genome will be useful to those engaged in research on the phylogenetic position of *A. kayasanensis* and the evolution of the genus *Adenophora*.

The genus *Adenophora*, commonly called ‘Radix Adenophora’, is an important plant resource as a source of herbal medicines (Ji et al. 2010). This genus, which belongs to Campanulaceae, is a perennial herbaceous plant genus with approximately 50–100 species that are distributed in temperate regions in Eurasia (Cheon et al. 2017). Among *Adenophora* species*, Adenophora kayasanensis* Kitam., discussed in this study, is a species endemic to Korea and was first described by Kitamura (1936) after its collection from Mt. Kaya National Park in Korea. In previous phylogenetic studies (Kim and Yoo 2011, 2012), the phylogenetic relationships of *A. kayasanensis* were not clear because it exhibited unresolved paraphyly with related taxa. In this study, we report the complete chloroplast genome sequence of *A. kayasanensis* for the first time, providing useful genetic information for further studies of the phylogenetic position of *A. kayasanensis* and the evolution of the genus *Adenophora*.

Plant materials of *A. kayasanensis* were collected from Mt. Kaya (37°28′17′′N, 130°54′03.77′′E), Hapcheon-gun, Gyeongsangnam-do province in South Korea, and a voucher specimen and DNA sample were deposited as Kangwon National University Herbarium (voucher no. KWNU93474; KS Cheon, cheonks@sangji.ac.kr). The total genomic DNA was extracted from *A. kayasanensis* leaves using a DNeasy Plant Mini Kit (Qiagen Inc., Valencia, CA) and paired-end (PE) libraries were prepared using a TruSeq DNA Sample Prep Kit HT (Illumina Inc, San Diego, CA) with a 350 bp average insert size. PE libraries were sequenced on a MiSeq platform (Illumina). We obtained 8,205,714 raw reads with a length of 301 bp. Low-quality sequences (Phred score < 20) were trimmed using CLC Genomics WorkBench version 6.04 (CLC Inc., Arhus, Denmark). After trimming, the library for *A. kayasanensis* included 6,539,849 reads. Then, *de novo* assembly was implemented using the Geneious prime^®^2021.1.1 (Biomatters Ltd, Auckland, New Zealand) and a total of 170,095 reads were aligned. We also compared each gene to the published complete chloroplast genome sequence of *Adenophora* species for correct gene annotation.

A typical quadripartite structure of *A. kayasanensis* is a DNA molecule 169,433 bp in length with 37.7 GC content, composed of a large single copy (LSC) region of 123,110 bp, a small single copy (SSC) region of 29,085 bp, and two inverted-repeat (IR) regions of 8619 bp. The plastid genome contains a total of 112 unique genes consisting of 78 protein-coding genes, 30 transfer *RNA* (*tRNA*) genes, and four ribosomal *RNA* (*rRNA*) genes. Of which, two genes contain two introns, and 15 genes contain one intron.

Phylogenetic analyses were conducted with 76 protein-coding genes of 14 accessions. Thirteen Campanulaceae *s. str.* species were selected as the ingroups, and one species (*Lobelia chinensis*) was chosen as the outgroup. The sequences were aligned using MAFFT (Katoh and Standley 2013). A maximum-likelihood (ML) analysis was performed using RAxML version 7.4.2 with 1000 bootstrap replicates and the GTR + I + Γ model (Stamatakis 2006). The ML tree formed the following two clades: platycodonoids and campanuloids. The campanuloids formed two subclades: the *Campanula s*. *str*. clade and the *Rapunculus* clade. All nodes in the ML tree were strongly supported, with 100% bootstrap value (BP) (Figure 1). Furthermore, Genus *Adenophora* forms a monophyletic and *A. kayasanensis* has a closer relationship with *A. racemosa* than with *A. divaricata*. In the light of the results of this study, therefore, it is judged that the taxonomic identity of *A. kayasanensis* will become clear if in-depth studies on the taxonomic relationships with *A. racemosa* are conducted in the future.

## Data Availability

The genome sequence data that support the findings of this study are openly available in GenBank of NCBI at (https://www.ncbi.nlm.nih.gov/) under the accession no. MZ365443. The associated BioProject, SRA, and bio-sample numbers are PRJNA736621, SRR14777593, and SAMN19655181, respectively. The ML tree for *Adenophora kayasanensis* based on 76 protein-coding genes. *Lobelia chinensis* (NC_035370) was used as outgroup. Numbers at nodes are bootstrap support values in %.
